# Artificial Intelligence Applied to a Robotic Dairy Farm to Model Milk Productivity and Quality based on Cow Data and Daily Environmental Parameters

**DOI:** 10.3390/s20102975

**Published:** 2020-05-24

**Authors:** Sigfredo Fuentes, Claudia Gonzalez Viejo, Brendan Cullen, Eden Tongson, Surinder S. Chauhan, Frank R. Dunshea

**Affiliations:** 1 Digital Agriculture, Food, and Wine Group, Faculty of Veterinary and Agricultural Sciences, The University of Melbourne, Parkville, VIC 3010, Australia; cgonzalez2@unimelb.edu.au (C.G.V.); eden.tongson@unimelb.edu.au (E.T.); 2 Agricultural Production System Modelling Group, Faculty of Veterinary and Agricultural Sciences, The University of Melbourne, Parkville, VIC 3010, Australia; bcullen@unimelb.edu.au (B.C.); ss.chauhan@unimelb.edu.au (S.S.C.); fdunshea@unimelb.edu.au (F.R.D.)

**Keywords:** machine learning, heat stress, animal welfare, climate change, automation

## Abstract

Increased global temperatures and climatic anomalies, such as heatwaves, as a product of climate change, are impacting the heat stress levels of farm animals. These impacts could have detrimental effects on the milk quality and productivity of dairy cows. This research used four years of data from a robotic dairy farm from 36 cows with similar heat tolerance (Model 1), and all 312 cows from the farm (Model 2). These data consisted of programmed concentrate feed and weight combined with weather parameters to develop supervised machine learning fitting models to predict milk yield, fat and protein content, and actual cow concentrate feed intake. Results showed highly accurate models, which were developed for cows with a similar genetic heat tolerance (Model 1: n = 116, 456; R = 0.87; slope = 0.76) and for all cows (Model 2: n = 665, 836; R = 0.86; slope = 0.74). Furthermore, an artificial intelligence (AI) system was proposed to increase or maintain a targeted level of milk quality by reducing heat stress that could be applied to a conventional dairy farm with minimal technology addition.

## 1. Introduction

Robotic dairy farms or Automated Milking Systems (AMS) are the result of the implementation of state of the art technology related to robotics to increase milk yield through increased efficiency and automation [1,2]. These technologies are developed in response to the increasing market opportunities for the dairy industry globally, which is projected to grow by 35% by 2030 [3]. However, global demands will also be accompanied by 14 million traditional dairy farms shutting down production due to increased competitiveness and requirements for guaranteed milk quality and animal welfare [4]. The latter is considered a growing concern for consumers, which is achieved by AMS since it is based on the “milking when they like” system increasing wellbeing and welfare of cows [5]. Further potential advances to AMS technologies have been researched in recent years through the implementation of biometrics monitoring of animals to assess physiological changes in production systems [6]. Some of these technologies are noninvasive using visible (RGB) imagery/video, and infrared thermal imagery for heart rate, respiration rate, and body temperature assessments. These technologies could result in improvements in the monitoring of heat stress in farm animals.

Modeling heat stress in AMS has concentrated recently on the rumination and milking performance [7], identifying specific thresholds with production factors [8] and thermal comfort indices [9], mainly through the calculation of the temperature-humidity index (THI) using several models [10]. According to a study by Nascimento et al. [11], who compared nine different models to calculate THI, the equation from Berman et al. [12] (Equation (10) below) was significantly correlated with physiological data of cows such as respiration rate, heart rate, rectal, and skin temperatures. However, all previous methods use deterministic mathematical equations with minimal animal information in the analysis, and the noncontact biometric analysis could be cost-prohibitive for the near-future application to conventional dairy farms.

Artificial intelligence (AI) applied to Digital Agriculture deals with the implementation and integration of digital data, sensors, and tools on agricultural applications from the farm to consumers [13]. These technologies can include big data, sensor technology, sensor networks, remote sensing, robotics, and unmanned aerial vehicles (UAV). Data processing is performed using new and emerging technologies, such as computer vision, machine learning, and AI, among others. The implementation of AI not only should benefit high technological systems, such as AMS, but also conventional dairy farms to increase their competitiveness in the future.

This research was based on machine learning modeling using ubiquitous environmental data obtained from automatic meteorological stations and cow information available by all dairy farms as inputs. Target information related to important parameters related to milk productivity, milk quality, and actual feed of dairy cows was obtained from an AMS belonging to The University of Melbourne, Australia. High accurate machine learning (ML) models that can be applied to any dairy farm from AMS to conventional were obtained. Furthermore, this paper proposes an AI system model to be implemented in any dairy farm to automatically assess and ameliorate heat stress by implementing ML models developed with an automated sorting and gate system.

## 2. Materials and Methods

### 2.1. Site, Robotic Dairy Farm, and Data Acquisition 

The study was conducted in a dairy farm located at The University of Melbourne Dookie College, Victoria, Australia (36°22’48” S, 145°42’36” E). This region had an average annual rainfall of 537 mm (monthly extremes: 30.5–57.6 mm) and mean daily solar exposure of 17 MJ m^2 −1^ (extremes: 7.3–27.3 m^2 −1^) from 1991–2019; data obtained from the Bureau of Meteorology (BoM) Dookie Agricultural College station 081013. The farm consists of 43 ha of irrigated pastures based on perennial ryegrass (Lolium perenne) and annual ryegrass (Lolium multiflorum). The herd in this site consists of Holstein-Friesian cows. The farm contains three Lely Astronaut robotic milking machines (Lely Holding S.à.r.l., Maassluis, The Netherlands), with a capacity of 60 cows per machine (maximum capacity of 180 cows) that move voluntarily for milking. As described by Dunshea et al. [14], cows wear an identification transponder neck collar (Lely Holding S.à.r.l., Maassluis, The Netherlands), which records the cows’ activity. The robotic milking system can automatically record parameters such as lactation days counted from day 0 at calving up to the time of next calving including the dry cow period, lactation number, milking frequency per day, milk yield (kg day^−1^), milk protein (%), milk fat (%) and somatic cells, programmed concentrate feed (kg day^−1^), concentrate feed intake (kg day^−1^), and liveweight (kg). Records of these data from June 2016 to March 2019 were used for this study.

Weather data were obtained from the meteorological station (Adcon Telemetry GmbH, Klosterneuburg, Austria), located at the Dookie Agricultural College, which provides data every 15 min for each day of the year. Parameters obtained were (i) temperature (*T*; °C), (ii) relative humidity (RH; %), (iii) rainfall as daily running total (mm), (iv) wind speed (km h^−1^), and (v) wind direction (°). Based on these data, other variables such as dewpoint temperature (*T_dp_*; °C; Equation (1); [15]), wet bulb temperature (*T_wet_*; °C), and THI were calculated. The latter was calculated using the following nine different equations (Equations (2)–(10); [11]):

(1) $$T_{dp}=\frac{243.5\;\left(\frac{17.67\times T}{243.5+T}+ln\frac{RH}{100}\right)}{17.67-\left(\frac{17.67\times T}{243.5+T}+ln\frac{RH}{100}\right)}$$

(2) $$THI_{1}=0.4\times \left(T+T_{wet}\right)\times 1.8+32+15$$

(3) $$THI_{2}=\left(0.15\times T+0.85\times T_{wet}\right)\times 1.8+32$$

(4) $$THI_{3}=\left(T\times 0.35+T_{wet}\times 0.65\right)\times 1.8+32$$

(5) $$THI_{4}=0.72\times \left(T+T_{wet}\right)+40.6$$

(6) $$THI_{5}=\left(1.8\times T+32\right)-\left[\left(0.55-0.0055\times RH\right)\times \left(1.8+T-26\right)\right]$$

(7) $$THI_{6}=\left(0.55\times T+0.2\times T_{dp}\right)\times 1.8+32+17.5$$

(8) $$THI_{7}=T+\left(0.36\times T_{dp}\right)+41.2$$

(9) $$THI_{8}=\left(0.8\times T\right)+\left(\frac{RH}{100}\right)\times \left(T-14.4\right)+46.4$$

(10) $$THI_{9}=3.43+1.058\times T-0.293\times RH+0.0164\times T\times RH+35.7$$

where *T_wet_* was calculated in batch using a customized code written in MATLAB® R2020a (Mathworks Inc., Natick, MA, USA; [16]), calculations were based on *T*, *T_dp_*, and surface pressure and the bisection search method.

### 2.2. Statistical Data and Machine Learning Modeling

Mean values of *THI* calculated with Equation (10) along with milk yield, milk protein, and fat content, and concentrate feed intake were obtained and plotted to visualize the effects of the different seasons on each parameter. Statistical data obtained from the inputs and targets consisted of minimum, maximum, and mean values of each parameter. 

**Figure 1:**
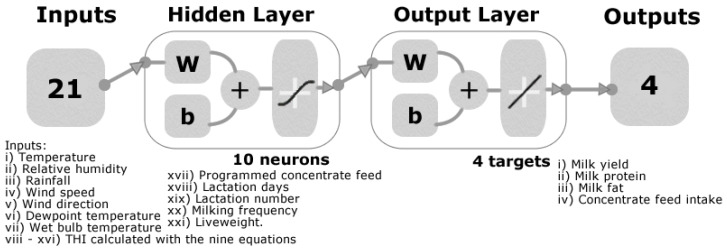
Diagram of the two-layer feedforward regression models with a tan-sigmoid function in the hidden layer and linear transfer function in the output layer. Abbreviations: THI: Temperature-humidity index; W: Weights; b: Bias.

Two ML models were developed based on artificial neural networks (ANN) using the Bayesian Regularization training algorithm. The latter was chosen as it showed the best accuracy and performance as well as no over or underfitting [17] after testing 17 different algorithms using a customized code written in MATLAB® R2020a. The inputs for the models (Figure 1) were based on the maximum values per day of the weather data (i) *T*, (ii) *RH*, (iii) rainfall, (iv) wind speed, (v) wind direction, (vi) *T_dp_*, (vii) *T_wet_*, (viii–xvi) *THI* calculated with the nine equations, and some data obtained from the robotic milking system, (xvii) programmed concentrate feed, (xviii) lactation days, (xix) lactation number, (xx) milking frequency, and (xxi) liveweight. The targets were also obtained from the robotic milking system. They consisted of (i) milk yield, (ii) milk protein, (iii) milk fat, and (iv) concentrate feed intake (i.e., cereal grain-based pellets fed to cows during milking, making up approximately 40% of cows diet). All data were normalized from −1 to 1. Model 1 was constructed using the data of cows with a similar heat tolerance (N = 36; heat tolerance range: 93–112) determined by estimation of Australian genomic breeding values for heat tolerance [18] following genotyping of each cow using hair follicle samples as per the commercial procedure (CLARIFIDE for dairy, Zoetis Australia Pty Ltd, Banyo, QLD, Australia). The genotyping experiment was approved by the University of Melbourne Faculty of Veterinary and Agricultural Science (FVAS) Animal Ethics Committee (AEC ID 1814645.1). In general, for heat stress, cows with Australian breeding values < 100 are less tolerant to hot, humid conditions than the average, while the cows with values > 100 are more tolerant than the average. Specifically, cows with breeding values of 93 will be 7% less heat tolerant than an average cow, and a cow with heat tolerance breeding values of 110 would be 10% more heat tolerant as compared to an average cow. In contrast, Model 2 was developed using data from all cows (N = 312) independent of their heat tolerance to create a general model. Samples were divided randomly as 70% for training and 30% for testing using a default derivative function. Ten neurons were chosen as the best number giving the highest accuracy and best performance based on the means squared error (MSE).

## 3. Results

**Figure 2:**
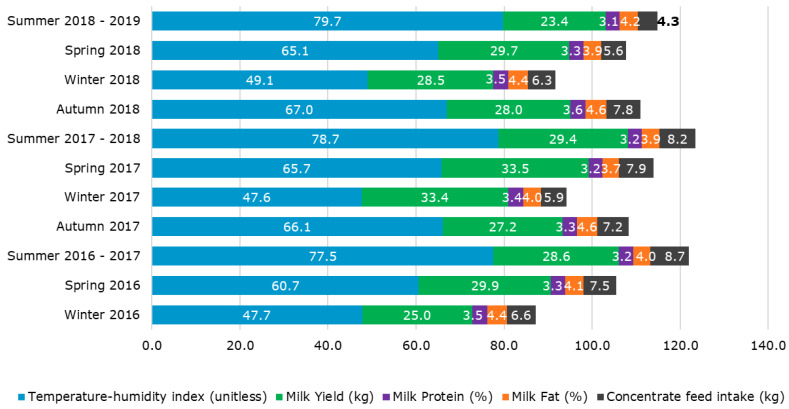
Mean values per season of each year for temperature-humidity index (*THI*_9_) and the four parameters used as targets in the machine learning (ML) models to represent the effect of different weather patterns on potential heat stress, milk productivity, and quality.

Figure 2 shows the mean values per season of each year for *THI*_9_ and the four parameters used as targets in the ML models to represent the effect of different weather patterns on those variables. As expected, the highest *THI* were obtained in the summer seasons of all years (77.5–79.7) and the lowest in winter of all years (47.6–49.1). The highest average milk yield per cow was observed in winter (33.4 kg day^−1^) and spring 2017 (33.5 kg day^−1^) with the lowest yield in summer 2018–2019 (23.4 kg day^−1^). The latter season also presented the lowest protein content in milk (3.1%) and concentrate feed intake (4.3 kg day^−1^). Spring 2018 and autumn 2018 had the lowest (3.9%) and the highest milk fat content (4.6%), respectively.

Table 1 shows the minimum, maximum, and mean values per year of each parameter used as inputs to construct the ML models. The lowest mean temperature (19.3 °C) was observed during 2016, which, at the same time, presented the lowest mean *THI*_1_–*THI*_9_ (58.1–72.0), highest mean *RH* (95.6%), and daily rainfall (3.9 mm). On the contrary, 2019 had the highest maximum temperature (44.9 °C) and, until March, the lowest mean *RH* (69.2%), and daily rainfall (0.3 mm), as well as the highest mean *THI*_1_–*THI*_9_ (68.6–82.8). Data for lactation days = 0 are the day the calf was born, and milk production commenced. Due to the voluntary milking system on the farm, there are some days when cows are not milked (i.e., milking frequency = 0). Furthermore, there were cows on the farm with extended lactations (>600 days). These were ‘carryover’ cows that were in an extended lactation because they failed to get pregnant in a timely manner.

Table 2 shows the minimum, maximum, and mean values of the parameters used as targets for the ML models. It can be observed that 2017 presented the highest milk yield per cow on average (30.7 kg day^−1^), although 2016 had the highest maximum milk yield per cow (65.4 kg day^−1^). Likewise, for milk protein, 2017 had the highest maximum value (6.1%), while 2018 presented the highest mean value (3.4%). Regarding milk fat content, 2019 had the highest maximum and mean values (10.9% and 4.3%, respectively). In 2019, the lowest average concentrate feed intake (4.0 kg day^−1^) was observed, while 2017 presented the highest mean (7.4 kg day^−1^) and the highest maximum value (24.3 kg day^−1^).

**Figure 3:**
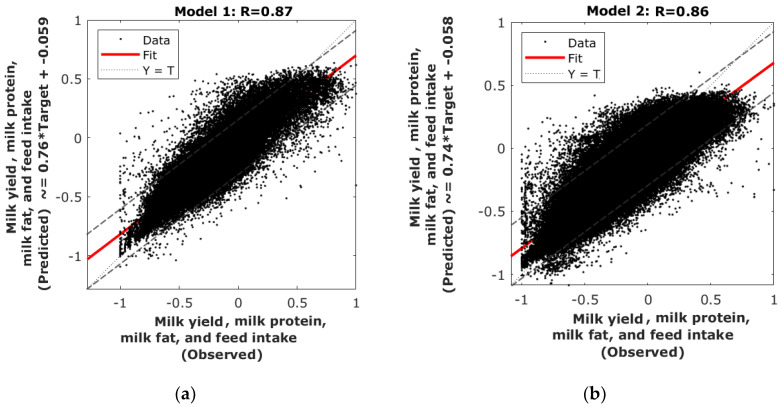
Overall regression graphs of (**a**) Model 1: Using the 36 cows with similar heat tolerance (93–112), and (**b**) Model 2: Using data from 312 cows.

Table 3 shows the statistical results of both models to predict milk yield, milk fat, and protein content, and concentrate feed intake. It can be observed that both models presented similar results with high overall correlation coefficients (Model 1: R = 0.87; Model 2: 0.86; Figure 3). None of the models showed any signs of overfitting as the correlation coefficient of all stages was the same, and the performance of training (Model 1: MSE = 0.0186; Model 2: MSE = 0.0154) was lower than the testing stage (Model 1: MSE = 0.0189; Model 2: MSE = 0.0157). According to the 95% confidence bounds, Model 1 presented 3.88% outliers (4513 out of 116,456) and Model 2 presented 3.60% (23,998 out of 665,836).

## 4. Discussion

### 4.1. Seasonality and Milk Yield

During the four years included in this study (2016–2019), there was a clear variation within seasons reflected by environmental parameters (*THI*) and milk productivity parameters (Figure 2). Higher heat stress risks for cows were observed in the summer of 2018–2019. Even though the *THI* parameter had a higher tendency, it was not significantly greater compared to the *THI* of summers belonging to 2017 and 2016 (*THI* = 79.7 compared to 78.7 and 77.5, respectively). However, milk yield and quality parameters were lower for 2018 compared with previous years. The high variability among all parameters shown through the years considered for this study can be considered as an advantage for ML modeling. These differences can be further supported by the data presented in Table 1 and Table 2 with more specific data per year. Prolonged periods of high temperature and relative humidity have shown to be detrimental to dairy cows performance due to heat stress [19]. This makes more critical the development of cost-effective methodologies to measure and alleviate heat stress during these periods of high *THI* [20]. 

### 4.2. Machine Learning Models

By investigating thermotolerance in cows from a genetic point of view, it could help to decrease economic losses associated with lower milk productivity, quality, and animal welfare [21,22]. Other methods have been based on the physical modification of the environment, such as shade and shelters, and dietary interventions to reduce heat stress effects, such as grape residue [23], açai [24], betaine [14,25], slowly fermentable grains [26], and other types of feed [27,28]. 

The ML models developed in this research (Model 1 and Model 2) do not differ much when considering 36 genetically similar cows for heat tolerance compared to a total of 320 cows. There is a slight difference in the slope for the general model considering all cows (Model 2; slope = 0.74) compared to Model 1 (slope = 0.76). Considering highly heat stress-tolerant cows helps to decrease underestimations made by Model 1 compared to Model 2. However, it can be considered that these differences are minimal when considering the number of cows deemed for Model 1 (n = 36) compared to Model 2 (n = 312). Furthermore, Model 1 presented a slightly higher percentage of outliers, considering them as outside the 95% confidence bounds, with 3.88% compared to 3.60% for Model 2 (Figure 3), this difference is minimal and small for both models considering the number of observations in each model (Table 3).

### 4.3. Artificial Intelligence to Manage Heat Stress and Milk Productivity

**Figure 4:**
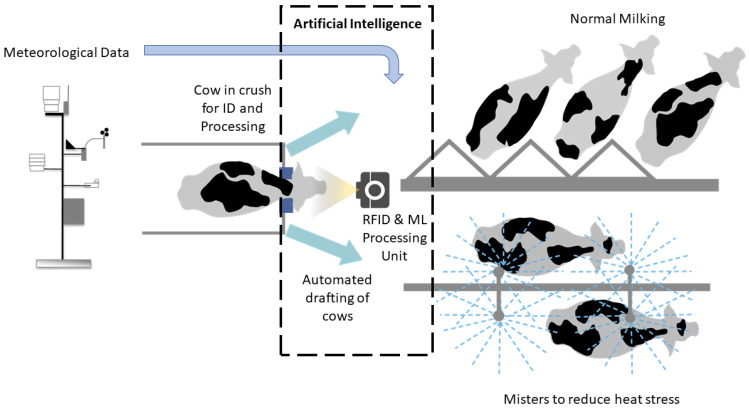
Proposed artificial intelligence (AI) application based on the automated processing of meteorological station and radio frequency identification system (RFID) for specific cow data input and machine learning (ML) processing. This system activates the gate system to draft cows to a cooling system or normal milking.

Physical modification of the environment to reduce ambient temperature or increase heat loss from the animal body, such as shading and fans, have been previously applied for lactating buffaloes with positive results [29], and in dairy cows using mixed-flow fans [30]. However, one of the most effective methods found is spraying water over animals using sprinkler systems [31,32,33,34]. This paper proposed the implementation of Model 2 with an automated system based on an individual cow assessment combined with environmental factors obtained from an automatic meteorological station (AME) (Figure 4). The AME can be easily connected to a processing unit (microprocessor or smartphone App) that can read the RFID from cows that are going to be milked to obtain cow information required by the model (Figure 1). The model outputs can be automatically set to specific thresholds for volume and milk quality that is desired by the dairy farm. The system can then automatically control gates to direct individual cows either to a cooling system with water sprinklers, the cows to reduce heat stress or to normal milking sections. The heat-stressed cows will be assessed the next day again, if they continue to be heat stressed, they will go to the sprinkler system and get milked to avoid mastitis.

The technical advantages of the proposed system (Figure 4) are: (i) ML modeling is based on readily available environmental data by most of the dairy farms and from government services with meteorological stations close to the farms; (ii) the environmental data can be automatically extracted from government services, such as the Bureau of Meteorology (BoM, Australia) [35] or by direct connectivity of a nearby automatic meteorological station to the RFID & ML Processing Unit (Figure 4); (iii) the digital database per cow can be implemented as part of the system to incorporate data such as programmed concentrate feed, lactation days and number, milking frequency, and liveweight. This information will need to be updated by the dairy farm personnel; (iv) cows can be identified by the system with normal RFID systems to extract cow data automatically from databases, and (v) the system requires an automated gate system to draft cows to the heat stress sprinkler system or the normal milking facilities. 

The managerial advantages that could be obtained by implementing the system proposed are: (i) milk volume and quality information available in real-time, per cow, and according to daily environmental conditions; (ii) prediction of actual concentrate feed intake per cow for feed monitoring management compared to programmed concentrate feed; (iii) real-time information to manage heat stress in a per cow basis to increase efficiency and maintain milk volumes and quality set as objectives, and (iv) data recorded from specific dairy farms can be incorporated in the model to increase the accuracy of target predictions.

With these considerations, an AI system for dairy farms can be implemented with reasonable investment affordable to small and medium dairy farmers. An alternative or complementary approach to an engineering solution may be to introduce dietary interventions such as betaine or antioxidants to cows likely to experience heat stress [14,28]. However, the time lag before the tissue concentrations of these nutrients are optimized could reduce the immediacy of this approach.

It should be noted that individual pasture intake could not be included in the model as the cows grazed as a single herd, so it was not measured. While this could no doubt add precision to the model, individual pasture intake cannot be measured under commercial grazing systems, and inclusion in the model would reduce its commercial utility. 

## 5. Conclusions

The machine learning models developed in this research may be applied to assess automatically animal welfare, milk productivity, and quality. Based on the inputs of the models, this machine learning modeling technique can be applied to any dairy farm. Implementation of Artificial Intelligence in dairy farms and the ML models developed here will require minimal technological additions, automated gate, and cooling systems. This paper has shown a practical application of AI using detailed information from a robotic dairy farm for the benefit of small and medium dairy farms to increase competitiveness in an increasingly demanding international market.

## Figures and Tables

**Table 1:** Minimum, maximum, and mean values of the parameters used as inputs to develop the machine learning models.

Parameter/Year	2016 *				2017				2018				2019 *			
	Min	Max	Mean	SD	Min	Max	Mean	SD	Min	Max	Mean	SD	Min	Max	Mean	SD
***T* (°C)**	7.9	37.8	19.3	7.15	8.3	42.0	22.2	8.27	8.9	43.3	22.6	7.90	16.3	44.9	31.7	6.07
***RH* (%)**	66.0	100	95.6	6.20	56.2	100	92.3	9.27	44.2	100	87.9	11.67	39.2	92.6	69.2	12.13
***T_dp_* (°C)**	3.8	24.9	11.6	3.40	2.1	22.8	11.5	3.97	1.6	22.1	10.2	3.75	4.3	21.2	13.5	3.79
***T_wet_* (°C)**	6.6	25.3	13.8	3.56	6.8	25.3	14.7	4.20	5.9	24.5	14.1	3.97	11.3	24.1	18.6	3.11
**Rainfall (mm day^−1^)**	0.0	34.0	3.9	6.76	0.0	31.6	1.86	4.55	0.0	37.8	1.4	4.07	0.0	5.0	0.3	0.99
**Wind speed (km h^−1^)**	5.2	38.3	15.1	5.22	5.8	34.3	15.3	5.19	5.1	38.0	16.2	5.82	9.4	39.8	19.5	6.14
**Wind direction (°)**	127.7	360.0	344.0	26.54	247.2	360.0	345.1	23.76	112.2	360.0	341.8	32.81	241.7	360.0	338.9	30.62
***THI*_1_**	57.2	89.6	70.6	7.37	58.4	94.6	73.3	8.72	58.7	92.9	73.3	8.34	67.3	96.3	82.8	6.25
***THI*_2_**	44.5	78.6	58.1	7.08	45.4	81.2	60.2	8.42	44.5	79.2	59.6	8.01	54.9	80.5	68.6	6.06
***THI*_3_**	44.7	81.1	60.0	8.24	46.0	86.8	62.9	9.79	46.3	83.8	62.6	9.34	56.3	87.8	73.2	6.99
***THI*_4_**	50.8	83.2	64.2	7.37	52.0	88.2	66.9	8.72	52.3	86.5	66.9	8.34	60.9	89.9	76.4	6.25
***THI*_5_**	47.1	82.2	63.4	8.15	47.5	86.5	66.5	9.13	49.0	84.3	66.9	8.48	60.4	87.5	76.2	5.55
***THI*_6_**	59.1	91.5	72.0	7.33	60.2	97.0	74.7	8.75	60.5	94.4	74.6	8.39	68.7	98.7	84.3	6.56
***THI*_7_**	50.8	83.6	63.9	7.40	51.9	89.1	66.6	8.84	52.2	86.5	66.5	8.47	60.6	90.9	76.3	6.62
***THI*_8_**	47.1	82.0	63.4	8.09	47.5	86.3	66.5	9.07	49.0	84.0	66.8	8.41	60.4	87.2	76.0	5.49
***THI*_9_**	33.4	86.6	58.8	12.45	33.4	92.6	63.7	13.73	36.5	89.4	64.4	12.61	55.4	93.7	78.1	7.83
**Programmed concentrate feed (kg day^−1^)**	0.0	15.0	8.9	3.03	0.0	23.0	8.5	3.11	0.0	15.7	7.8	3.15	0.0	8.0	5.1	2.30
**Lactation number**	1.0	6.0	2.7	0.97	1.0	7.0	3.0	1.24	1.0	7.0	2.3	1.61	1.0	8.0	3.0	1.75
**Lactation days**	0.0	736.0	225	158.17	0.0	668.0	198.1	139.28	0.0	705.0	228.3	142.26	0.0	755.0	227.5	144.01
**Milking frequency (per day)**	0.0	5.0	2.4	0.71	0.0	6.0	2.5	0.75	0.0	6.0	2.4	0.84	0.0	5.0	1.9	0.81
**Liveweight (kg)**	373.0	938.0	677.7	82.85	428.0	951.0	668.2	78.25	335.0	959.0	655.4	84.57	410.0	896.0	629.5	71.86

* Values from 2016 cover from June to December and 2019 cover from January to March. Abbreviations: Min: Minimum; Max: Maximum; *T*: Temperature; *RH*: Relative humidity; *T_dp_*: Dewpoint temperature; *T_wet_*: Wet-bulb temperature; *THI*: Temperature-humidity index; SD: Standard deviation.

**Table 2:** Minimum, maximum, and mean values of the parameters used as targets to develop the machine learning models.

Parameter/Year	2016 *			2017			2018			2019 *		
	Min	Max	Mean	Min	Max	Mean	Min	Max	Mean	Min	Max	Mean
**Milk yield (kg day^−1^)**	0.0	65.4	28.1	0.0	60.2	30.7	0.0	61.2	28.8	0.0	52.1	21.2
**Milk protein (%)**	1.8	5.8	3.3	1.8	6.1	3.2	2.2	5.8	3.4	0.9	4.9	3.1
**Milk fat (%)**	1.0	10.7	4.2	0.8	10.2	4.0	0.7	10.3	4.2	0.7	10.9	4.3
**Concentrate feed intake (kg day^−1^)**	0.0	19.5	7.3	0.0	24.3	7.4	0.0	18.8	6.7	0.0	10.6	4.0

* Values from 2016 cover from June to December and 2019 cover from January to March. Abbreviations: Min: Minimum; Max: Maximum.

**Table 3:** Statistical results of each stage of the machine learning models.

Stage	Samples (Cows x Days)	Observations (Samples x Targets)	R	b	Performance (MSE)
**Model 1**					
**Training**	20,380	81,520	0.87	0.76	0.0186
**Testing**	8734	34,936	0.86	0.76	0.0189
**Overall**	29,114	116,456	0.87	0.76	-
**Model 2**					
**Training**	116,521	466,084	0.86	0.74	0.0154
**Testing**	49,938	199,752	0.86	0.74	0.0157
**Overall**	166,459	665,836	0.86	0.74	-

Abbreviations: R: Correlation coefficient; b: Slope; MSE: Means squared error.
